# Fracture-driven weakening amplifies projected ice loss from West Antarctica

**DOI:** 10.1073/pnas.2601529123

**Published:** 2026-07-06

**Authors:** Javier Blasco, Violaine Coulon, Maaike Izeboud, Thomas Gregov, Yanjun Li, Frank Pattyn

**Affiliations:** ^a^ Laboratoire de Glaciologie, Department of Geosciences, Environment, Society, Université libre de Bruxelles, Brussels 1050, Belgium; ^b^Earth System Complexity Group, Department of Polar Terrestrial Environmental Systems, Alfred-Wegener-Institute, Helmholtz Centre for Polar and Marine Research, Potsdam 14473, Germany; ^c^ Department of Water and Climate, Vrije Universiteit Brussel, Brussels 1050, Belgium; ^d^ Institute of Earth Surface Dynamics, Faculty of Geosciences and the Environment, Université de Lausanne, Lausanne CH-1015, Switzerland; ^e^ School of Atmospheric Sciences, Southern Marine Science and Engineering Guangdong Laboratory, Sun Yat-sen University, Zhuhai 519082, China

**Keywords:** Antarctica, ice-sheet modeling, damage, sea level rise

## Abstract

Ice shelves buttress the flow of the Antarctic ice sheet, but this stabilizing effect weakens as fractures form, reducing ice viscosity and accelerating ice discharge toward the ocean. Here, we incorporate physically consistent damage mechanics into an ice-sheet model and apply it across the Amundsen Sea Embayment, the largest current contributor to Antarctic mass loss. Simulations reproduce key satellite-derived damage patterns under present-day conditions. Projections to 2300 show that allowing damage to evolve strongly amplifies future ice loss, increasing sea-level contributions by up to a factor of ∼4.5. Even when damage is held fixed at its present-day extent, projected contributions increase by 50 to 130%, indicating that existing fractures already commit the system to enhanced ice loss. Basal crevasses dominate this response, accounting for ∼90% of damage-induced amplification. Healing mechanisms substantially moderate damage, as neglecting gravitational crevasse closure and compressive healing overestimates ice loss by almost 50%. Together, these results suggest that fracture-driven weakening substantially alters Antarctic ice-sheet dynamics and should be represented in projections of future sea-level rise.

## Significance

Most projections of Antarctic ice loss omit fracture mechanics. Using an ice-sheet model that explicitly represents ice damage, we show that fractures accelerate ice flow and amplify sea-level contributions from West Antarctica by up to 4.5-fold by 2300. Even when no new fractures form, existing cracks commit the system to 50 to 130% more ice loss than projections assuming undamaged ice. Basal crevasses, which penetrate deeply into ice shelves, dominate this response by weakening ice more effectively than surface fractures. At the same time, gravitational closure of crevasses substantially limits damage growth. Together, these results demonstrate that fracture-driven weakening significantly alters ice-sheet dynamics and must be represented to reliably project future Antarctic contributions to sea-level rise.

The Antarctic Ice Sheet is potentially the largest yet most uncertain contributor to future sea-level rise (1). The West Antarctic Ice Sheet is particularly vulnerable to marine ice-sheet instability (2), whereby ice grounded on retrograde bed slopes is prone to rapid and sustained grounding-line retreat through a positive feedback. The onset of such instability is closely linked to ice-shelf stability (3). Although ice shelves do not directly contribute to sea-level rise, they regulate ice discharge by buttressing the ice sheet, slowing inland ice velocities (4). When ice shelves thin or weaken, their buttressing capacity is reduced, accelerating inland ice flow and increasing ice flux across the grounding line, causing further retreat (5).

Satellite observations reveal that regions with high strain rates, such as ice shelves and grounded ice near grounding lines, are particularly prone to crevasse formation (6–8). Fractures form when the effective stress applied to the ice exceeds its failure strength, causing it to crack due to its brittle nature (9–11). These cracks accumulate as damage, which influences ice rheology by reducing viscosity, thereby enhancing ice flow and promoting further crevasse formation and structural weakening through a positive feedback loop (6, 8). Fractures can also trigger additional weakening processes, such as hydrofracturing (12), surface loading by dolines (13), surface depressions associated with basal crevasses (14, 15), and ice-front calving (16). Consequently, fracture formation effectively weakens ice shelves and may ultimately lead to their disintegration. Using a data-inferred relationship between damage and ice flow characteristics applied to existing ice-sheet model projections, Izeboud et al. (8) predict increased damage development, particularly under high-emission climate scenarios. However, this approach does not account for the influence of evolving damage on ice dynamics. Capturing such two-way interactions requires coupling ice-sheet models with a damage model.

Previous studies have adopted various approaches to represent damage in ice-sheet models, but not all allow for fully coupled interactions between damage and ice dynamics. One common method infers damage through inversion techniques, by adjusting ice-flow parameters to match observed surface velocities (7, 17–19). While this approach closely captures present-day conditions, it cannot predict how damage will evolve under changing climate and stress regimes, and therefore assumes that damage remains constant over time. Forward modeling approaches, in contrast, simulate the physical processes driving damage evolution, enabling a two-way coupling with ice-sheet dynamics. Fracture mechanics theories, including zero-stress and linear elastic fracture mechanics (LEFM), have been widely used to understand crevasse propagation (16, 20–22). Zero-stress models determine crevasse depth where tensile stress balances lithostatic pressure (20, 21), whereas LEFM predicts crack propagation based on stress intensity factors. However, these approaches are challenging to implement into continental-scale ice-sheet models due to resolution constraints and the mismatch between crack formation and ice flow timescales (23). Continuum damage mechanics (CDM) addresses these limitations by representing damage as a continuous field variable rather than tracking individual fractures, enabling efficient integration into large-scale ice-sheet models. Several studies have implemented CDM models to estimate the ratio of fractured ice depth to total ice thickness while representing damage as a spatially varying field (6, 24–27). Other CDM implementations adopt nonlocal formulations or phase field methods coupling fracture mechanics with poromechanics to simulate hydrofracturing (28, 29), or employ anisotropic theory for creep fracture (19, 30–33).

While most CDM applications have focused on the link between damage and calving, fewer studies have examined how damage-induced weakening influences ice viscosity and flow acceleration (6, 23, 24, 27, 34, 35). Applications beyond idealized geometries remain limited (27, 36, 37), and most studies have treated damage as a diagnostic quantity rather than a dynamically evolving component of the ice-sheet system (25, 26). Here, we use the Kori-ULB ice flow model, which incorporates damage initiation and evolution through a CDM zero-stress formulation (20), to assess the influence of damage-induced weakening on the ice flow and dynamics in the Amundsen Sea Embayment (ASE), Antarctica. The ASE is a region of rapid mass loss (38) and a major potential contributor to future sea-level rise due to its susceptibility to marine ice-sheet instability. Projections suggest that this area may contribute the largest Antarctic ice loss in the coming decades and centuries (39–43), primarily driven by enhanced warm-water intrusions (44). This region has also experienced increased crevasse formation since the 2010s (6–8, 37). Although recent studies suggest limited buttressing from the Thwaites Ice Shelf (45), the effect of crevasse formation in this region has been shown to enhance ice mass loss and grounding-line retreat (27).

Dynamically evolving damage was recently applied to Thwaites Glacier using a similar modeling framework (27), but damage was allowed to develop from an initially undamaged state, without accounting for the accumulated damage expected under present-day stress conditions. Here, we present an improved damage formulation that accounts for gravitational crevasse closure and compressive healing mechanisms and initialize an ice-sheet model with physically consistent damage across the Amundsen Sea Embayment. By both initializing with observationally consistent damage and allowing it to evolve dynamically over time, our framework bridges the gap between inversion-based methods, which infer present-day damage but assume it remains constant, and previous prognostic applications that neglect preexisting damage. We evaluate multiple spin-up configurations against satellite observations, examine the impact of damage under schematic future forcing, and perform sensitivity analyses to identify the dominant controls on damage evolution and its feedbacks on ice flow.

## Results

### Present-Day Configurations.

We perform five spin-up experiments, in which the model is run to a present-day quasi-equilibrium under fixed climate forcing while nudging toward present-day ice-sheet geometry (*Materials and Methods*), using different damage configurations: 1) undamaged, 2) full damage (both surface and basal crevasses), 3) surface-only damage, 4) basal-only damage, and 5) full damage but neglecting gravitational crevasse closure and compressive healing ($S_{0}=1$ in Eq. **11**). RMSE in ice thickness remain below 50 m across all experiments, with differences concentrated near the Thwaites Glacier grounding line, reflecting small variations in grounding-line position. Velocity RMSE exceeds 200 m y^−1^ but is reduced when damage is included (*SI Appendix*, Fig. S1). Velocity SDs are the largest across the Pine Island and Thwaites ice shelves.

**Fig. 1.**
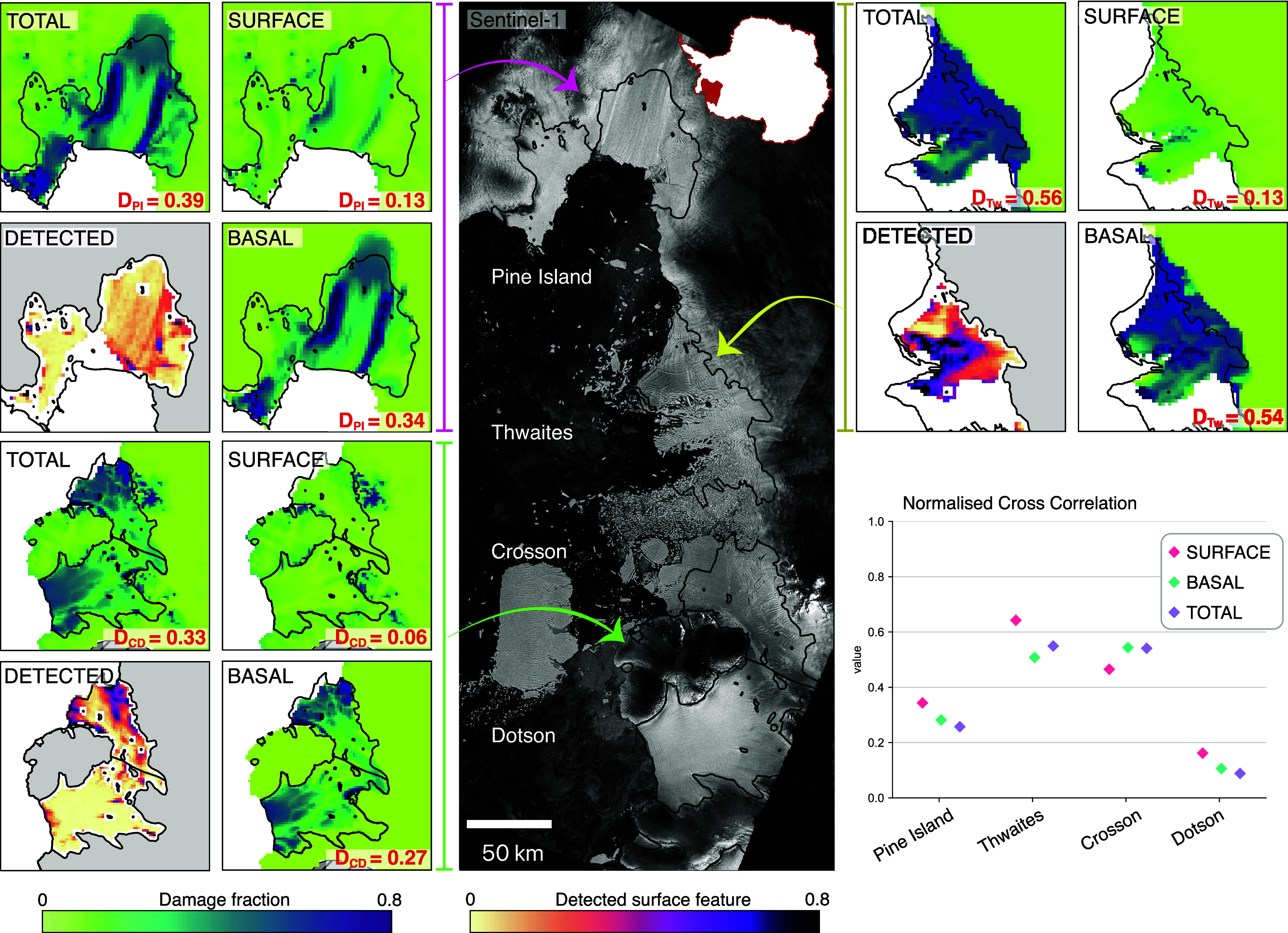
Observed and simulated present-day damage patterns in the Amundsen Sea Embayment. *Center* panel: Sentinel-1 GRD images of September 2019, which were (including other years) used to derive damage. Surrounding panels: Present-day simulated damage patterns for Pine Island (*Top Left*), Thwaites (*Top Right*), and the Crosson–Dotson ice shelves (*Bottom Left*). For each ice shelf, the four subpanels correspond to: total damage (*Top Left*), surface-damage-only (*Top Right*), and basal-damage-only (*Bottom Right*). Red numbers indicate mean damage values for the corresponding ice shelf. *Bottom Left* panel shows the detected surface features signal for 2015–2020, derived from ref. 8. Because the satellite-derived damage signal reflects surface fracture features visible in imagery while the modeled damage represents depth-averaged crevasse penetration, the two quantities are not directly comparable in magnitude; different colormaps are therefore used to highlight spatial patterns rather than absolute values. *Bottom-Right* panel: Metric (best score is 1) used to identify the closest match between the observed and simulated damage fields for each ice shelf.

Simulated damage fields (Fig. 1 and *SI Appendix*, Fig. S2 for case 5) show that damaged areas are concentrated across ice shelves and near ice fronts. Thwaites Glacier Ice Shelf exhibits the most widespread weakening, with a spatially averaged damage value of 0.56, compared with 0.39 for Pine Island and 0.33 for Crosson-Dotson ice shelves. Pine Island Ice Shelf displays enhanced damage along its shear margins, while the Crosson-Dotson ice shelves show stronger damage toward their calving front. Those patterns are consistent with satellite-derived damage estimates (6, 8). Basal crevasses are the dominant contributor to the overall weakening: Surface-only damage reduces the mean ice-shelf damage by a factor of 3 to 5 relative to the full-damage configuration, while basal-only values are close to the combined case. Across all experiments, damage remains below the imposed threshold of 0.8 (*Materials and Methods*).

Normalized cross-correlations (which measure the agreement in spatial patterns between images, invariant to shifts in intensity and contrast) with the satellite-derived damage signal over ice shelves for 2015–2020 (8) (Fig. 1) indicate that surface crevasses generally reproduce the detected spatial patterns better than basal-only fields. This suggests that many features identified by the detection algorithm, such as near-surface crevasses, rifts, and fracture zones, may be more strongly influenced by surface processes, even though basal weakening may still play a critical role in ice-shelf stability. The Crosson Ice Shelf is however an exception: Here, basal damage aligns more closely with the detected signal, suggesting that damage in this region may be more strongly controlled by basal processes. This interpretation is consistent with (46), which suggested that surface features in this region may be influenced by basal processes. These comparisons should nevertheless be interpreted with caution as i) damage is a highly dynamic field that evolves rapidly in response to changing stress conditions, and ii) the two quantities are not directly comparable: the satellite-derived detected signal of refs. 8 and 47 reflects the visual expression of fracture features at the surface (based on grayscale contrast of linear structures in satellite imagery) rather than a direct measure of crevasse depth.

### The Effect of Damage on Future Ice Loss.

**Fig. 2.**
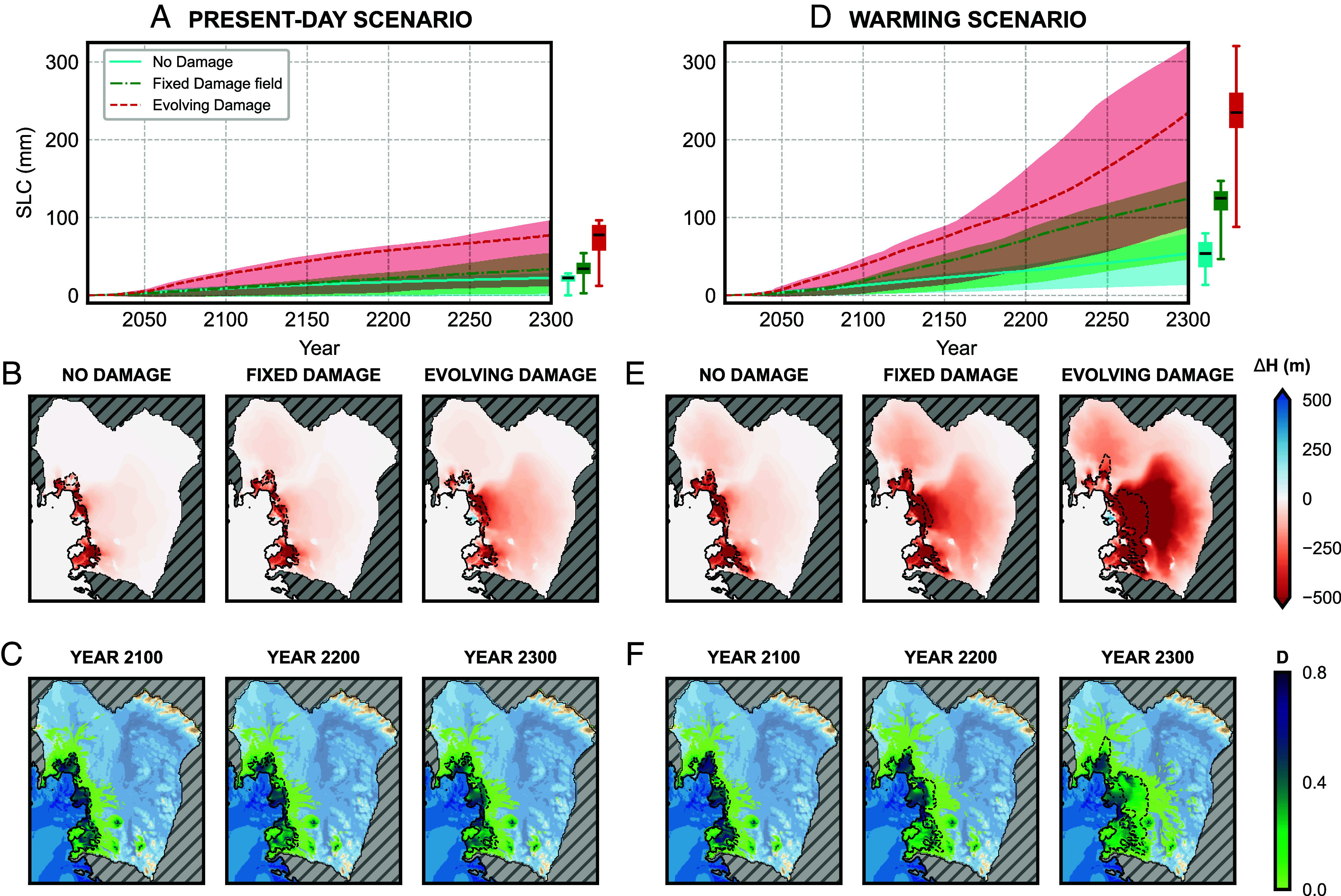
Damage-driven weakening enhances ice loss under present-day and warming conditions. (A) Sea-level contribution (SLC) under constant present-day forcing for three model configurations: undamaged ice (blue), fixed damage (green), and evolving damage (red). Solid lines represent the ensemble mean; shaded regions indicate the full ensemble range (minimum to maximum). Boxplots show the interquartile range, with whiskers extending to the minimum and maximum of the ensemble. (B) Mean ice-thickness anomaly between 2300 and 2015 under constant present-day forcing. Dashed black lines show the most advanced and retreated grounding-line positions across the ensemble. (C) Snapshots of mean damage evolution at years 2100, 2200, and 2300 for the evolving-damage case under constant present-day forcing. (D) As in A, but for an idealized warming scenario. (E) As in B, but for an idealized warming scenario. (F) As in C, but for an idealized warming scenario.

We extend the present-day spin-up configurations to 2300 under both constant present-day forcing and an idealized ocean-warming scenario reaching +2.5 °C (48) (*SI Appendix*, Fig. S3), comparable to SSP5-8.5 projections for the Amundsen Sea Embayment (49), using a 50-member ensemble exploring uncertainties in ice dynamics and ice–ocean interactions (*Materials and Methods*). These simulations show that damage strongly amplifies the projected mass loss (Fig. 2), indicating that damage mechanics, that are currently missing from most ice-sheet projections, may exert a significant influence on future ASE evolution.

Even when the damage field is held fixed at its present-day (spin up) state, mass loss substantially increases. This shows that existing structural weakening accelerates ice flow and enhances ice loss, consistent with (6). By 2300, the ensemble-mean mass loss increases by a factor of 1.5 relative to undamaged ice under constant present-day climate (from 22.4 mm to 34.2 mm of sea-level rise equivalent; see *SI Appendix*, Table S1), and by a factor of 2.3 under schematic ocean warming.

Allowing damage to evolve dynamically amplifies mass loss much further, supporting the positive feedback mechanism suggested by previous modeling studies (6, 7) and observations (8) whereby damage-enhanced flow promotes further damage formation. By 2300, dynamically evolving damage increases mass loss by a factor of 3.5 (constant present-day climate) and 4.4 (ocean warming) compared to undamaged ice. This higher sensitivity underscores the nonlinear response of the ASE to warming when accounting for the structural weakening of the ice. The ensemble spread also increases substantially, reflecting a wider range of projected grounding-line positions when damage evolution is permitted (*SI Appendix*, Fig. S4). The enhanced projected ice loss when accounting for ice damage results from more extensive grounding-line retreat, particularly at Thwaites Glacier under warming, where retreat penetrates farther inland than in the undamaged or fixed-damage cases.

The spatial evolution of the damage field differs substantially between forcing scenarios (Fig. 2). Under constant present-day conditions, damage remains relatively stable with limited inland propagation, suggesting that the current ASE configuration can maintain near-steady-state grounding-line positions despite existing structural weaknesses. For a warming scenario, however, grounding-line retreat initiates a positive feedback loop (8): Higher strain rates drive further damage development, which propagates inland, leading to ice-shelf structural weakening, speed-up, and further retreat.

### The Relative Contributions of Surface and Basal Damage.

**Fig. 3.**
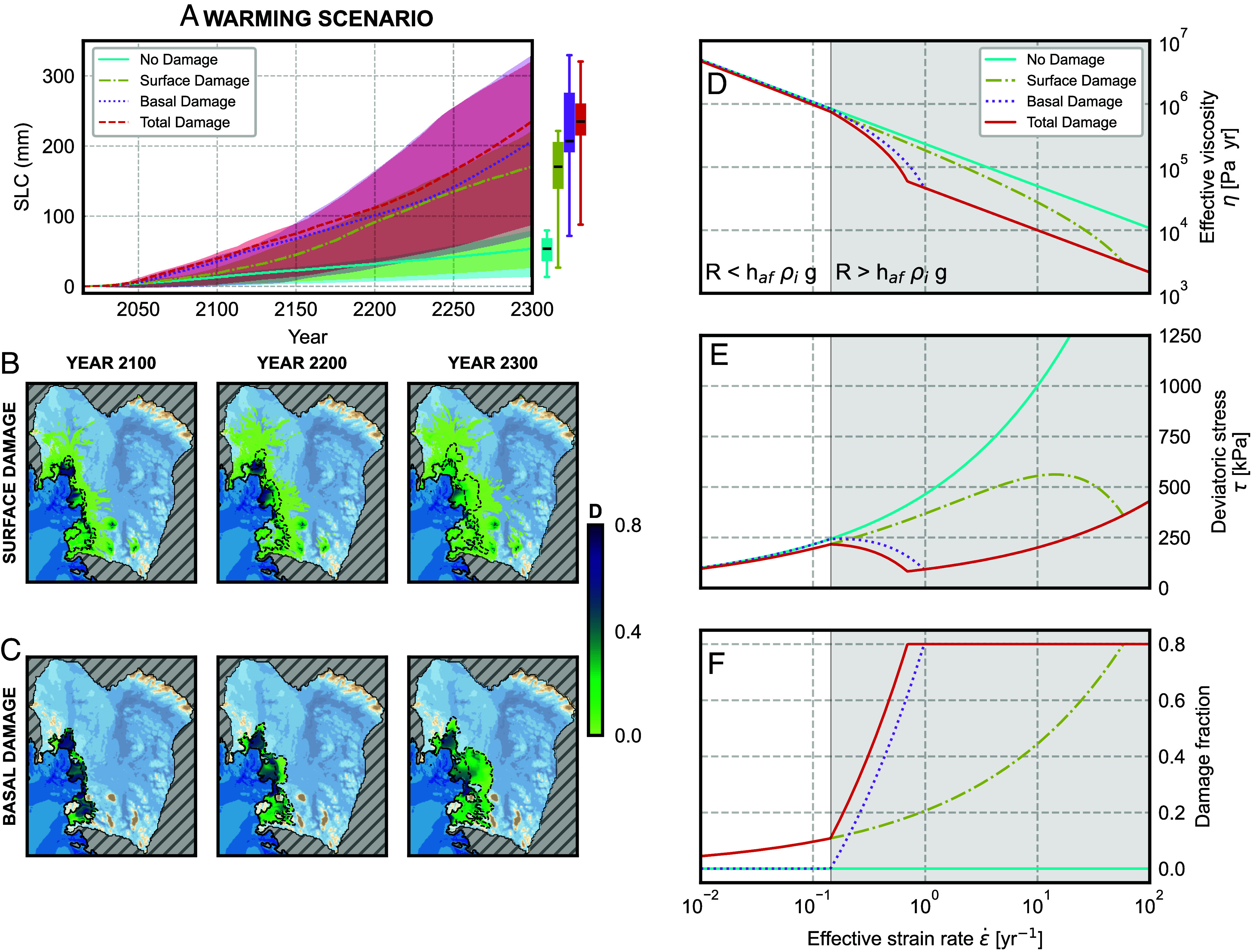
Relative contributions of surface and basal damage to ice-sheet weakening. *Left* panels: Sea-level contribution under an idealized warming scenario for (*A*) undamaged ice (blue), surface-damage-only (yellow), basal-damage-only (purple), and combined surface and basal damage (red). The solid lines represent the ensemble mean, while the shaded regions indicate the full ensemble range (minimum to maximum). Boxplots show the interquartile range, with whiskers extending to the minimum and maximum of the ensemble. *Middle* and *Lower* panels: Snapshots of the mean damage-field evolution at years 2100, 2200, and 2300 for the surface-damage experiment (*B*) and the basal-damage experiment (*C*). *Right* panels: Idealized (*D*) effective viscosity η; (*E*) deviatoric stress τ; and (*F*) damage fraction D for an undamaged case (blue line), combined-damage case (red line), surface-damage only case (yellow line), and basal-damage only case (purple line). For the visualization, an ice thickness of 500 m and a flow factor 10^−17^ Pa^−3^ y^−1^ is considered. The gray band indicates the strain-rate range where resistive stress (R) exceeds the height above flotation ($h_{\mathrm{af}}$).

Sensitivity experiments in which only surface or basal damage is allowed to evolve isolate the respective contributions of surface and basal crevasses to the projected ice loss (Fig. 3*A*). Basal damage exerts the dominant control on damage-induced weakening and flow acceleration: By 2300, the basal-only damage experiment produces ∼90% of the mass loss obtained in the full surface-basal configuration (207 mm mean sea-level rise equivalent), whereas the surface-only case reaches ∼72% (170 mm). Both mechanisms greatly increase mass loss compared to the undamaged configuration (54 mm). Both surface- and basal-damage experiments also exhibit substantially larger ensemble spreads than the undamaged case, driven by more variable grounding-line retreat trajectories across ensemble members (*SI Appendix*, Fig. S4).

The spatial patterns of the two damage mechanisms differ markedly: Surface crevasses produce lower damage values, but damage propagates farther inland on the grounded ice (Fig. 3*B*), whereas basal crevasses yield higher damage magnitudes but remain confined to regions where resistive stresses exceed the buoyancy pressure of the ice above flotation (Fig. 3*C*), which is a prerequisite for their initiation (Eq. **10**).

The physical basis for the dominance of basal damage becomes evident when examining its behavior under idealized ice-shelf conditions (500 m ice thickness and a flow rate factor $A=10^{-17}$ Pa^−3^ y^−1^; Fig. 3*D*–*F*). At low to moderate strain rates, resistive stress remains below the buoyancy pressure threshold; only surface crevasses can form, but their depths remain around 10% of the ice thickness, implying limited impact on effective viscosity and local stress (Fig. 3*D*–*F*). Once strain rates increase sufficiently for resistive stress to exceed the height above flotation, basal crevasses initiate and grow rapidly. The upper damage limit of 0.8 is reached at strain rates nearly two orders of magnitude lower for basal crevasses than for surface crevasses (Fig. 3*F*). This occurs because basal crevasses penetrate deeper into the ice column before tensile stresses are balanced by lithostatic pressure, and seawater pressure within the crevasse promotes further opening (Eq. **10**). In contrast, surface crevasses are limited by atmospheric pressure. As a result, basal damage produces much stronger ice-shelf rheological weakening, leading to greater ice flow acceleration.

Despite its weaker influence relative to basal damage, surface damage alone still yields roughly three times the mass loss of the undamaged case by 2300 (170 mm versus 54 mm sea-level equivalent), indicating that even modest damage magnitudes can substantially alter ice dynamics. The combined effect of surface and basal crevasses accelerates ice-shelf weakening beyond what either component produces individually, although the additional contribution beyond basal damage alone remains modest.

### Damage Healing and Compression Regimes.

As crevasses are advected with the ice flow, several processes can lead to their healing, i.e., the closure of crevasses. These include compressive closure in regions of convergent flow and gravitational overburden (50), refreezing of meltwater within crevasse cavities (51), erosion of crevassed undersides by subshelf melting, and burial of surface fractures by snow accumulation (24). Because many of these processes remain poorly constrained and occur at spatial scales not resolved by ice-sheet models, we focus on mechanisms that can be directly related to the resolved stress and mass-balance fields. Our formulation therefore accounts for crevasse opening and closure in response to compression, extensional thinning, and gravitational restoring forces (50), as well as healing associated with surface accumulation and basal melting (24) (*Materials and Methods*). To assess how different healing mechanisms influence damage evolution and projected ice loss, we compare two damage transport formulations: 1) a reference scheme that includes all healing mechanisms represented in our damage transport equation; and 2) a simplified formulation that neglects gravitational crevasse closure and compressive healing, accounting only for healing from surface accumulation and basal melting (24).

**Fig. 4.**
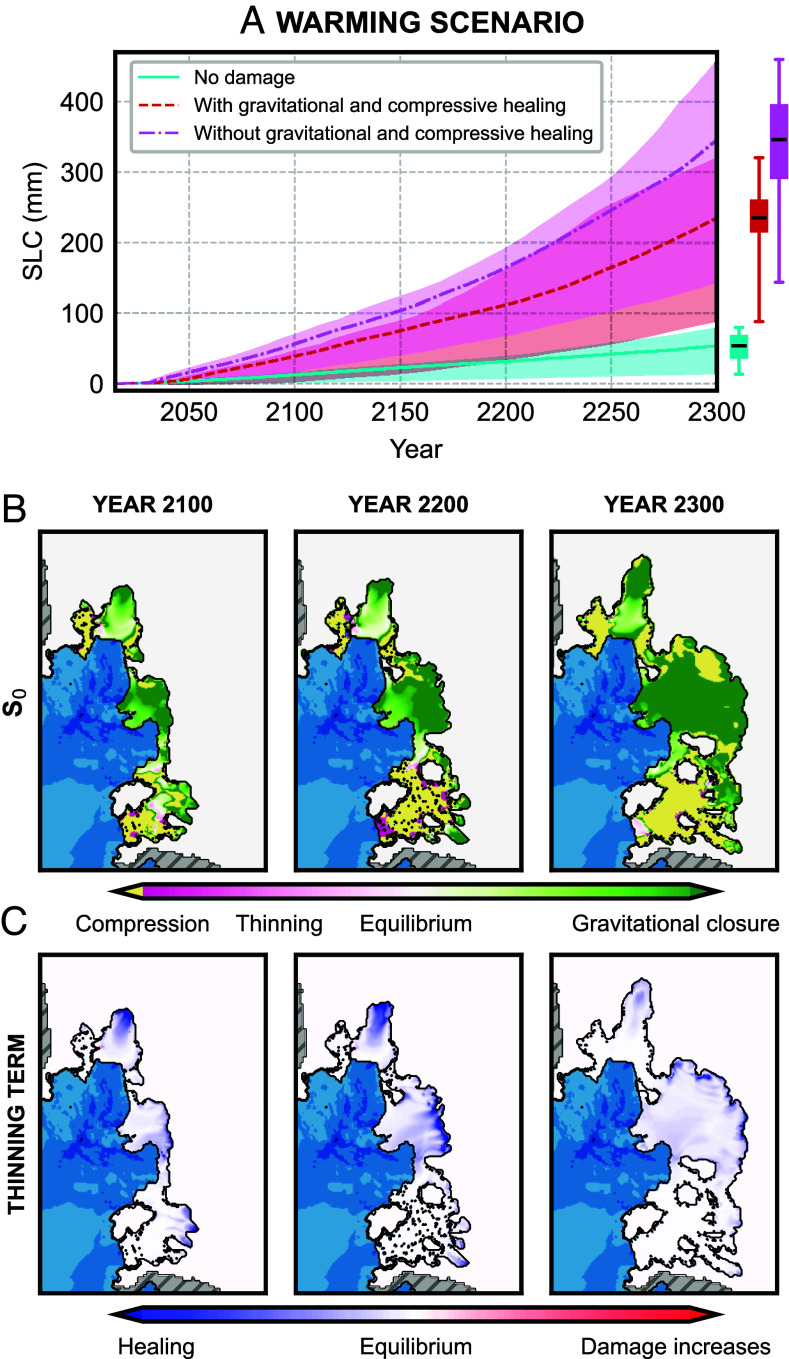
Role of gravitational and compressive healing in regulating damage-driven ice loss. Sea-level contribution under an idealized warming scenario for undamaged ice (blue), the reference formulation with gravitational and compressive healing (red), and the configuration without gravitational and compressive healing (pink) (*A*). The solid lines represent the ensemble mean, while the shaded regions indicate the full ensemble range (minimum to maximum). Boxplots show the interquartile range, with whiskers extending to the minimum and maximum of the ensemble. Snapshots at years 2100, 2200, and 2300 for the ensemble member that best matches the mean sea-level curve simulated with gravitational and compressive healing: (*B*) the ratio of hydrostatic pressure to first principal deviatoric stress ($S_{0}$), where $S_{0}>1$ indicates gravitational healing, $0<S_{0}<1$ extensional thinning, and $S_{0}<0$ compressive healing, and (*C*) the resulting damage thinning/healing term from the long-wavelength approximation.

Neglecting gravitational crevasse closure and compressive healing produces substantially larger projected ice loss: The ensemble-mean sea-level contribution increases by 47% relative to the reference formulation (Fig. 4*A*). Spatial damage fields also show markedly higher damage values across the ASE when these healing mechanisms are omitted (*SI Appendix*, Fig. S5), and grounded ice probabilities indicate a far more retreated grounding line position (*SI Appendix*, Fig. S4). This suggests that the additional healing mechanisms included in the reference formulation strongly constrain damage accumulation.

To understand where and why these healing mechanisms operate, we examine spatial patterns of the large-scale ratio between the hydrostatic pressure and the first principal deviatoric stress ($S_{0}$ in Eq. **14**, see *Materials and Methods*). The spatial distribution of $S_{0}$ (Fig. 4*B*) reveals distinct healing mechanisms: Strong compression ($S_{0}<0$) dominates the Crosson–Dotson ice shelves, while gravitational restoring forces ($S_{0}>1$) prevail across much of Pine Island and Thwaites. Consequently, most of the domain experiences net healing (Fig. 4*C*).

This explains the lower damage magnitudes obtained when gravitational restoring forces are accounted for: Despite local extension, gravitational pressure can dominate over deviatoric stresses, allowing crevasses to close under their own weight. This gravitational healing mechanism is especially important in thick ice-shelf regions, where hydrostatic pressure is large relative to tensile stresses. Because this process is absent from the simplified mass-balance-only formulation, it allows damage to accumulate more freely, leading to substantially greater ice loss.

## Discussion

The damage law used in this study follows continuum damage mechanics, in which crevasse depth depends on the deviatoric stress field through the Nye zero-stress approximation (16, 20, 21, 24, 50, 52), applied here in its original form without any stress threshold for damage initiation. Instead of capturing the initiation of individual cracks when tensile stresses exceed a critical threshold, typically ranging from 150 to 750 kPa(53–55), the Nye formulation assumes a stress balance for crevasse depth: A crevasse deepens until the tensile deviatoric stress at its tip is balanced by lithostatic pressure. This results in a depth-averaged crevasse density that captures the bulk weakening of the ice without explicitly resolving individual cracks, making the zero-stress formulation particularly convenient for continental-scale ice-sheet models. This approach is appropriate when crevasses are closely spaced (56), such that the influence of neighboring crevasses cancels out, leaving the intact ice between crevasses unaffected (57). Bassis et al. (58) recently confirmed numerically that Nye’s crevasse depth estimates remain accurate under these conditions, but may underestimate penetration depth when fracture spacing increases. The zero-stress model may therefore be interpreted as a lower bound (22). It also cannot capture some types of damage, such as large, isolated rifts or shear fractures. In addition, it is important to stress that the crevasses represented here correspond to meter-scale features resolved at 2 km grid resolution, whereas ice failure results from the progressive accumulation of microcracks at much smaller scales (19, 23, 28, 30, 33, 59). Capturing this multiscale nature of ice damage, from microscale grain boundary sliding to meter-scale crevasses to kilometer-scale rifts, remains a major challenge. Nonlocal formulations (31) or multiscale coupling approaches may be needed to capture the full range of relevant processes. A more detailed representation of individual fracture dynamics would require linear elastic fracture mechanics (LEFM), where crack propagation depends explicitly on stress intensity and ice fracture toughness. In our simulations, however, stresses exceeding 150 kPa are mostly confined to high-strain regions near grounding lines and shear margins (*SI Appendix*, Figs. S6 and S7). This suggests that even if a finite stress threshold for damage initiation were imposed (as done, for example, in ref. 23), damage would still concentrate in the dynamically critical regions where it has the strongest influence on ice discharge and grounding-line stability. Although real crevasses may not strictly conform to the zero-stress theory, the qualitative features of the formulation are still expected to hold. While different fracture models will have slightly different functional forms, they all remain functions of the stability number $S_{0}$ (58), suggesting that the qualitative dependence of damage on the stress state should be robust even if the precise functional relationship is uncertain. Moreover, the absence of tunable parameters in the formulation provides additional confidence that the model captures the dominant physics governing crevasse penetration, at least to first order. To better constrain the limitations of zero-stress models and improve confidence in sea-level projections, future studies should focus on benchmarking such parameterizations against higher-fidelity models, such as 3-D full-Stokes simulations or phase field fracture models.

In the meantime, comparison with satellite observations (8) provides an important evaluation of our parameterization. Our present-day configurations capture key observational patterns: enhanced damage along the shear margins of the Pine Island Ice Shelf, widespread damage across Thwaites Ice Shelf, and high damage near the Crosson-Dotson front. Surface damage correlates more strongly with the satellite signal, consistent with the fact that many damage features visible in satellite imagery, i.e., surface depressions, fracture textures, chaotic zones, originate from surface processes. While basal crevasses can also produce depressions at the ice shelves’ surfaces (14, 15), no continental-scale basal crevasses dataset currently exists. The weaker agreement with observations for simulations including basal damage suggests that many basal features important for ice-shelf weakening may not be captured by existing detection algorithms. The satellite-derived signal and the modeled damage, however, represent fundamentally different definitions of damage: The observations capture surface expressions of fractures visible in satellite imagery, whereas the model represents mechanical weakening through depth-averaged crevasse penetration. Their comparison should therefore be interpreted primarily as a qualitative test of whether the model reproduces the large-scale distribution of fractured regions rather than as a validation of simulated damage magnitudes. Overall, normalized cross-correlation values reach a maximum of approximately 0.6, indicating that the spatial agreement between modeled and observed damage remains imperfect. This mismatch suggests some uncertainty in the spatial distribution of the modeled damage-driven weakening and hence in the projected ice mass changes, particularly for the Dotson ice shelf, where correlation is the lowest.

In our simulations, climate forcing affects damage primarily through dynamic changes in the stress field. Although ice-shelf thinning reduces driving stresses, grounding-line retreat and flow acceleration increase strain rates near the grounding line, promoting fracture formation. The resulting damage is then advected downstream with the ice flow, allowing ice-shelf interiors to remain highly damaged even where local stresses would not generate new fractures (*SI Appendix*, Fig. S7). Healing mechanisms therefore exert a significant influence on the projected ice loss by limiting the downstream accumulation of damage. Neglecting healing from gravitational restoring forces (when hydrostatic pressure exceeds deviatoric stresses) and from compressive stresses increases ASE mass loss by 47% by 2300. Large sectors of the Pine Island and Thwaites ice shelves currently fall within this regime, despite being in extensional flow. Without these healing mechanisms, damage accumulates unrealistically in ice-shelf interiors, leading to excessive weakening and potentially overestimated mass loss projections. While basal melting in our formulation effectively acts as a healing mechanism by removing the damaged ice, direct observations reveal more complex behavior inside basal crevasses, with a mixture of freezing and melting depending on local ocean circulation and cavity geometry (51). In some cases, melting may accelerate crevasse propagation rather than heal it. Our mass balance-based healing term therefore represents a simplified assumption that likely yields lower-bound estimates of damage evolution.

Several processes not represented in our simulations could further affect damage evolution and ice flow. While we capture how damage-induced weakening affects flow acceleration, we do not simulate the role of damage on the formation and propagation of crevasses leading to calving. Instead, the ice-front position is held fixed. Imposing calving where damage exceeds a critical threshold (e.g., following 60) may reduce buttressing and potentially increase both damage and the projected ice loss, especially given that healing currently predominates in ASE ice-shelf interiors (Fig. 4*C*), allowing these ice shelves to maintain a buttressing capacity despite substantial damage. In addition, while subshelf melting is treated here as a healing mechanism through the erosion of the crevassed underside of ice shelves, observations of channelized melting beneath ice shelves suggest that localized melt channels may instead promote fracture growth and interact with damage along shear margins, potentially creating a positive feedback between melt channel formation and damage that is not captured here (61). In contrast, refreezing within basal crevasses has also been observed in ice-shelf cavities and may contribute to healing, but such thermodynamic processes are not explicitly represented in our model formulation. Similarly, the accumulation of icebergs and sea ice can lead to ice mélange formation and thickening at ice-shelf fronts, exerting a back pressure that resists fracture growth (16, 62) and may slow down damage propagation over short timescales. However, this stabilization process (also omitted here) is expected to weaken under long-term warming due to reduced sea ice and iceberg persistence (63). Because our goal is to isolate the role of damage mechanics on ice-sheet evolution rather than to produce comprehensive projections, we here prescribe an idealized, spatially uniform, ocean-warming scenario that does not include changes in surface melt or precipitation (*SI Appendix*, Fig. S3). Increased precipitation could enhance surface accumulation and partially offset damage, whereas surface melt would favor hydrofracturing, a process also not included in our simulations. Water-filled crevasses enhance fracture propagation through additional hydraulic pressure and can trigger ice-shelf collapse (12, 64), potentially amplified by the melt-elevation feedback (42). Future work should assess the effect of these interacting processes on damage under comprehensive climate projections.

## Conclusion

We present a comprehensive assessment of damage evolution in the Amundsen Sea Embayment using a continuum damage mechanics framework embedded in a continental-scale ice-sheet model, achieving stable present-day configurations that reproduce satellite-inferred damage patterns. We show that damage mechanics substantially amplify the projected ice loss from this sector (by up to a factor of ∼4.5 by 2300) through a positive feedback in which damage-enhanced flow promotes further crevasse formation. Even holding damage fixed at present-day values increases ice mass loss, indicating that existing fracture patterns already commit the system to accelerated retreat. Basal crevasses dominate this response, as seawater pressure allows deeper penetration and stronger effective ice weakening than surface crevasses alone. Healing mechanisms are critical for realistic damage projections. Neglecting healing from gravitational restoring forces and from compressive stresses overestimates mass loss by 47%. Our analysis reveals that large regions of Pine Island and Thwaites ice shelves experience gravitational restoring forces strong enough to close crevasses even under extensional flow, underscoring the need to represent both damage formation and healing to accurately capture ice-sheet dynamics. These findings have direct implications for sea-level projections and climate policy. Most ice-sheet models contributing to IPCC assessments lack explicit damage mechanics, potentially underestimating West Antarctic ice loss over the coming decades and centuries. The nonlinear amplification under warming, where damage accelerates flow, drives grounding-line retreat, and increases strain rates, hence promoting further damage, is a strong positive feedback. Given the vulnerability of the Amundsen Sea Embayment to marine ice-sheet instability, incorporating damage processes into ice-sheet models is essential for improving future projections, which are critical for robust risk assessment and adaptation planning.

## Materials and Methods

### Ice Flow Model.

The Kori-ULB ice flow model (42, 65, 66) is a vertically integrated, thermomechanical, hybrid ice-sheet/ice-shelf model. The flow of grounded ice is computed as the sum of the shallow-ice (SIA) and shallow-shelf (SSA) approximations, whereas ice flow of floating ice shelves is represented only by the SSA solution (67). The grounding-line position is determined from the flotation criterion.

Ice viscosity (η) is described by Glen’s flow law, expressed as

[1] $$\begin{array}{cc} \eta & =\frac{A{\left(T\right)}^{-\frac{1}{n}}}{2}{\left|\dot{\varepsilon }\right|}^{\frac{1}{n}-1}, \end{array}$$

where $A\left(T\right)$ is a temperature-dependent flow-rate factor that follows an Arrhenius relationship (68), and n is the Glen exponent typically set to 3. The thermomechanically derived viscosity represents the rheology of undamaged ice, while damage effects are incorporated separately (*Damage Transport, Healing, and Evolution*). Ice temperatures evolve through a heat conduction-convection equation for the ice column (69). Following the SSA, the strain rate $\dot{\varepsilon }$ has a magnitude given by

[2] $$\begin{array}{cc} |\dot{\varepsilon }| & =\sqrt{{\left(\frac{\partial u}{\partial x}\right)}^{2}+{\left(\frac{\partial v}{\partial y}\right)}^{2}+\frac{1}{4}{\left(\frac{\partial u}{\partial y}+\frac{\partial v}{\partial x}\right)}^{2}}, \end{array}$$

where u and v are the horizontal velocity components in the x and y directions, respectively.

The deviatoric stress (τ) is computed as the product of the ice viscosity and the strain rate:

[3] $$\begin{array}{cc} \tau & =2\eta \dot{\varepsilon }. \end{array}$$

Basal friction ($\tau_{\text{b}}$) is represented by a regularized Coulomb-friction law (70, 71):

[4] $$\begin{array}{c} \tau_{\text{b}}=A_{\text{b}}^{-\frac{1}{m}}{\left(\frac{u_{0}}{u_{\text{b}}+u_{0}}\right)}^{\frac{1}{m}}u_{\text{b}}^{\frac{1}{m}}, \end{array}$$

where m is the sliding index here set to 3, $u_{0}$ the velocity regularization term, and $A_{\text{b}}$ the sliding coefficient.

Present-day ice-sheet conditions are provided by a spin-up simulation nudging toward present-day ice-sheet geometry (42, 72, 73) from Bedmachine v3 (74). Basal sliding coefficients under grounded ice and subshelf melt rates under floating ice are adjusted iteratively to reduce the misfit with observed ice thickness. Under these balance melt rates, the model reaches a quasi-steady grounding-line configuration even when damage evolves dynamically, indicating that damage alone does not inevitably trigger grounding-line retreat. The present-day surface mass balance and temperature fields are prescribed from the regional climate model RACMO2.3p2 (75), while oceanic temperatures and salinity fields are taken from ref. 76.

In the forward simulations, melt beneath the floating ice shelves is calculated using the PICO model (77), based on the box model by ref. 78. PICO simulates the buoyancy-driven advection of ambient ocean water into the ice-shelf cavity up to the grounding line and then upward along the ice draft through consecutive boxes k. Basal-melt below ice shelves is computed as

[5] $$\begin{array}{c} {\mathrm{BMB}}_{k}=\gamma_{T}^{*}\left(\frac{\rho_{\mathrm{sw}}c_{p}}{\rho_{i}L_{i}}\right)\left(T_{k}-T_{f,k}\right), \end{array}$$

where $\rho_{\mathrm{sw}}$ and $\rho_{i}$ are the density of sea-water and ice, respectively; $L_{i}$ the latent heat of ice; $c_{p}$ the specific heat of sea-water; and $\gamma_{T}^{*}$ the effective heat exchange velocity. The maximum number of boxes is set to 10 for a better representation of basal melt rates pattern (*SI Appendix*, Table S2).

Basal melting beneath grounded ice is calculated from the difference between the local basal temperature gradient and the gradient corrected for pressure melting, using the geothermal heat flux from ref. 79.

### Damage Parameterization.

We define damage D as the ratio between the total fractured ice depth δh and the total ice thickness h:

[6] $$\begin{array}{cc} D & =\frac{\delta h}{h}=\frac{d_{\text{s}}+d_{\text{b}}}{h}, \end{array}$$

where $d_{\text{s}}$ and $d_{\text{b}}$ are the surface and basal crevasse depths.

Damage reduces the effective ice viscosity $\eta^{*}$ following

[7] $$\begin{array}{cc} \eta^{*} & =\left(1-D\right)\eta . \end{array}$$

Crevasse depths are computed using the zero-stress approximation, which assumes that crevasses propagate to the depth at which the resistive stress R equals the ice overburden pressure, also called lithostatic stress (20, 52):

[8] $$\begin{array}{cc} d_{\text{s}} & =\frac{R}{\rho_{i}g}, \end{array}$$

[9] $$\begin{array}{cc} d_{\text{b}} & =\frac{\rho_{\text{i}}}{\rho_{\text{sw}}-\rho_{\text{i}}}\left(\frac{R}{\rho_{i}g}-h_{\text{af}}\right), \end{array}$$

[10] $$\begin{array}{cc} R & =2\tau_{1}+\tau_{2}, \end{array}$$

where $h_{\text{af}}$ is the ice thickness above flotation. Following refs. 25 and 26, R includes both principal stresses, differing from formulations that only included the first principal stress (6, 23, 24, 27). We consider dry crevasses only, hence excluding any hydrofracturing effects from water-filled crevasses. Surface damage initiates at lower strain rates than basal damage, as the resistive stress must exceed the ice overburden pressure for basal crevasses to form.

### Damage Transport, Healing, and Evolution.

Damage evolution follows the long-wavelength formulation of ref. 50, which accounts for fracture thinning (necking) caused by the stretching and widening of crevasses under extensional strain:

[11] $$\begin{array}{cc} \frac{\partial D}{\partial t} & +\nabla \cdot \left(uD\right)=\left[n^{*}\left(1-S_{0}\right){\dot{\varepsilon }}_{1}-\frac{\dot{m}}{h}\right]D, \end{array}$$

[12] $$\begin{array}{cc} \dot{m} & =\;\max\left(\;\text{SMB},0\right)+\max\left(\;\text{BMB},0\right). \end{array}$$

Subshelf melting (BMB, positive values) reduces damage by removing the fractured layer, thereby eroding the crevassed underside, whereas accumulation increases the thickness of undamaged ice. Consequently, both surface and basal mass balance components contribute to healing. Their effects are capped by the respective crevasse depths to prevent overhealing, i.e., damage reduction beyond the existing fractured layer.

The term $n^{*}\left(1-S_{0}\right){\dot{\varepsilon }}_{1}$ represents the strain-induced thinning of existing fractures, which depends on the modified Glen exponent

[13] $$\begin{array}{c} n^{*}=\frac{4n\left(1+\alpha +\alpha^{2}\right)}{4\left(1+\alpha +\alpha^{2}\right)+3\left(n-1\right)\alpha^{2}}, \end{array}$$

where n=3 is the Glen’s flow law exponent and $\alpha =\varepsilon_{2}/\varepsilon_{1}$ is the ratio of the principal strain rates. The scaling factor $S_{0}$ represents the large-scale ratio between the hydrostatic pressure and the first principal deviatoric stress:

[14] $$\begin{array}{c} S_{0}=\frac{\rho_{\text{i}}\left(\rho_{\text{sw}}-\rho_{\text{i}}\right)gh}{2\tau_{1}\rho_{\text{sw}}}. \end{array}$$

The stress used to compute $S_{0}$ is taken from the previous timestep, meaning that it is evaluated using the stress field of the evolving damaged ice rather than that of purely undamaged ice. When $S_{0}>1$, gravitational restoring forces dominate and crevasses close (i.e., healing under the ice’s own weight). For $0<S_{0}<1$, extensional thinning dominates and crevasses grow due to tensile stress, leading to damage growth. For $S_{0}<0$, the first principal stress is negative, and crevasses heal due to compression (as ${\dot{\varepsilon }}_{1}$ is negative). Crevasses can therefore heal even in the absence of negative strain rates. Such strain-induced thinning is only applied to floating ice.

At each time step, the final damage field is defined as the maximum value between the transported damage and the newly formed local damage. This allows low-stress regions to inherit damage from upstream areas of higher stress. Because locally computed damage represents the instantaneous fracture response to the stress field while transported damage evolves through advection and healing terms, this formulation implicitly separates the short fracture timescale from the longer healing timescale associated with ice flow. To prevent numerical instabilities, we limit the damage fraction to a maximum of 0.8 (i.e., 80% of the total ice thickness), similar to ref. 23.

### Experimental Setup.

The model domain covers the drainage basins of Pine Island, Thwaites, and Crosson-Dotson glaciers, resampled to a horizontal resolution of 2 km. Resolution sensitivity tests (*SI Appendix*, Fig. S8) indicate that this resolution is close to numerical convergence for key model outputs. Damage is initialized during the model spin-up simulation (*Present-Day Configurations*), allowing the damage field to evolve through advection and healing until a quasi-steady distribution consistent with the present-day ice-sheet configuration is reached.

Projections to 2300 are based on a 50-member ensemble exploring uncertainties in ice dynamics and ice–ocean interactions. Using a Latin hypercube design, the ensemble samples the sliding-law regularization parameter ($u_{0}$, ranging from 100 to 500 m y^−1^), and the effective thermal exchange velocity controlling subshelf melting ($\gamma_{T}^{*}$, ranging from 10^−5^ to 10^−4^, see *SI Appendix*, Table S2). To ensure consistent initial conditions across experiments, we first optimize the sliding coefficients using $u_{0}=300\;$m y^−1^ as a reference. For each alternative $u_{0}$, the sliding coefficients are then rescaled so that the resulting basal friction and velocity fields remain comparable to the reference state.

Each ensemble member is run to 2300 under two climate forcings: 1) constant present-day climate conditions, and 2) idealized ocean-warming reaching +2.5 °C (48) (*SI Appendix*, Fig. S6), comparable to SSP5-8.5 projections for the Amundsen Sea Embayment (49).

To isolate the role of damage, we prescribe a fixed calving front and omit both the melt–elevation feedback and glacial isostatic adjustment.

### Comparison to Satellite-Derived Damage.

The satellite-derived damage signal is a pixel-wise median of annual damage maps between 2015 and 2020 from ref. 8. These maps were constructed from Sentinel-1 Synthetic Aperture Radar (SAR) ground range detected (GRD) images from the September–November period of each year using the Normalized Radon Transform Damage detection algorithm (47). The damage map is downsampled from 400 m spatial resolution to the ASE model grid of 2 km using bilinear resampling, and normalized from 0 to 0.5 to 0 to 1 value range.

The similarity between simulated and satellite-derived damage fields is evaluated using the Normalized Cross Correlation coefficient (NCC) (80), which measures spatial pattern agreement while being invariant to differences in intensity and contrast. This is crucial, as simulated damage values and satellite-derived signals differ in physical meaning: The latter reflects feature contrast in the satellite image (47) rather than actual fracture depth as represented by the damage parameter D. Consequently, standard metrics such as Mean Absolute Error (MAE) or Root Mean Squared Error (RMSE) are less appropriate for this comparison.

## Supplementary Material

Appendix 01 (PDF)

## Data Availability

Scripts and model outputs; Kori-ULB is maintained as a git repository data have been deposited in Zenodo and Github (DOI: 10.5281/zenodo.13645430; https://github.com/FrankPat/Kori-ULB; and https://zenodo.org/records/15358474) (81, 82).
