# Supplementary material for: Fracture-driven weakening amplifies projected ice loss from West Antarctica
Source: Proc Natl Acad Sci U S A. 2026 Jul 6;123(28):e2601529123. doi: 10.1073/pnas.2601529123 (PMC13367866; doi:10.1073/pnas.2601529123)
Supplement: Supplementary file 1 — Appendix 01 (PDF) [file pnas.2601529123.sapp.pdf]

Supporting Information for

The effect of ice damage on the glaciers of the Amundsen Sea Embayment, Antarctica

Javier Blasco, Violaine Coulon, Maaike Izeboud, Thomas Gregov, Yanjun Li, Frank Pattyn

Corresponding Authors:
E-mail: javier.blasconavarro@awi.de and violaine.coulon@ulb.be

This PDF file includes:

Figs. S1 to S8
Tables S1 to S2
SI References

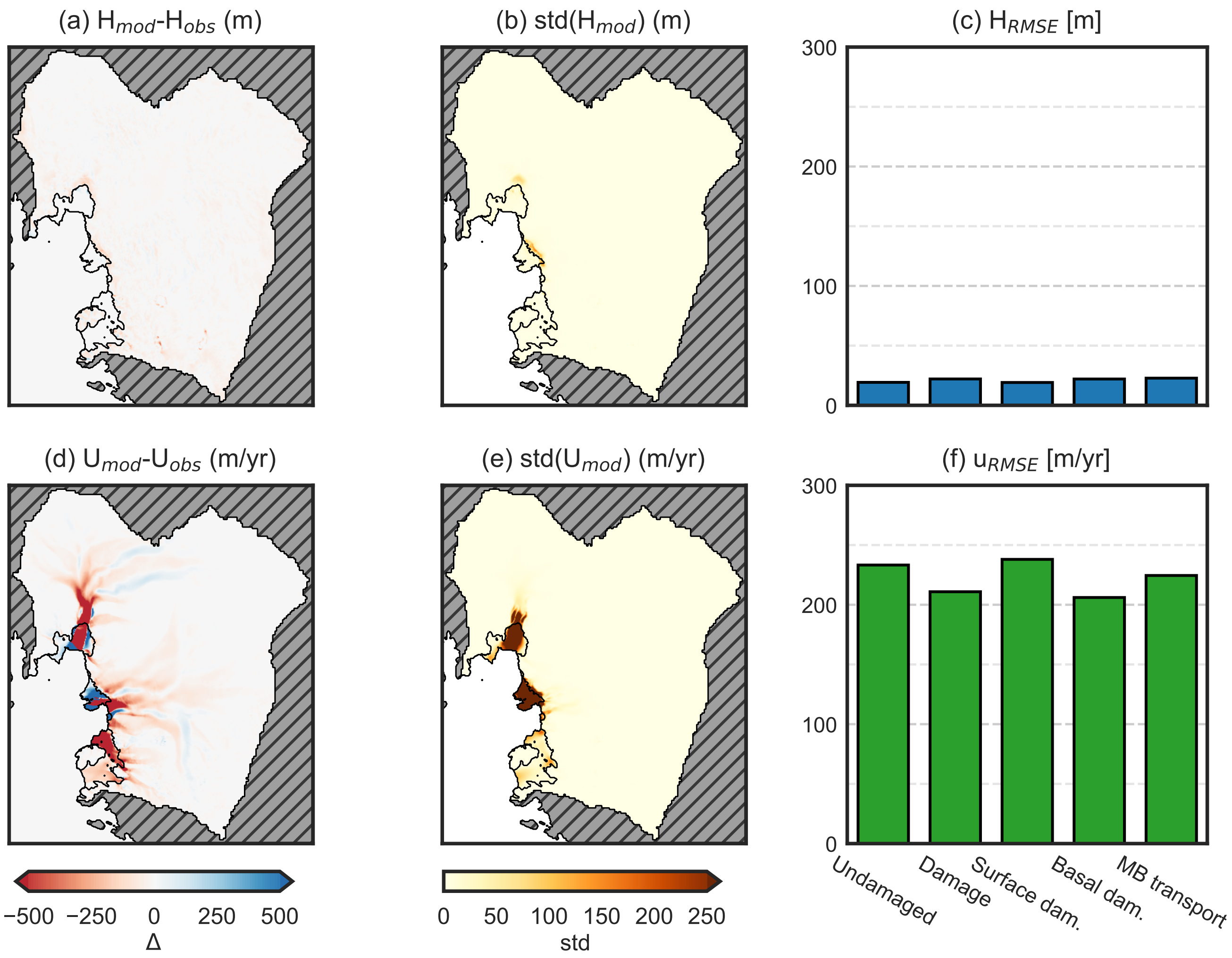

Fig. S1. Spinup diagnostics for the five-member ensemble. (a) Ice-thickness error of the ensemble mean relative to observations from (1). (b) Standard deviation of modeled ice thickness across ensemble members. (c) Root-mean-square error (RMSE) of modeled ice thickness for each ensemble member. (d) Surface-velocity error of the ensemble mean relative to (2). (e) Standard deviation of modeled surface velocity across ensemble members. (f) Root-mean-square error (RMSE) of modeled surface velocity for each ensemble member.

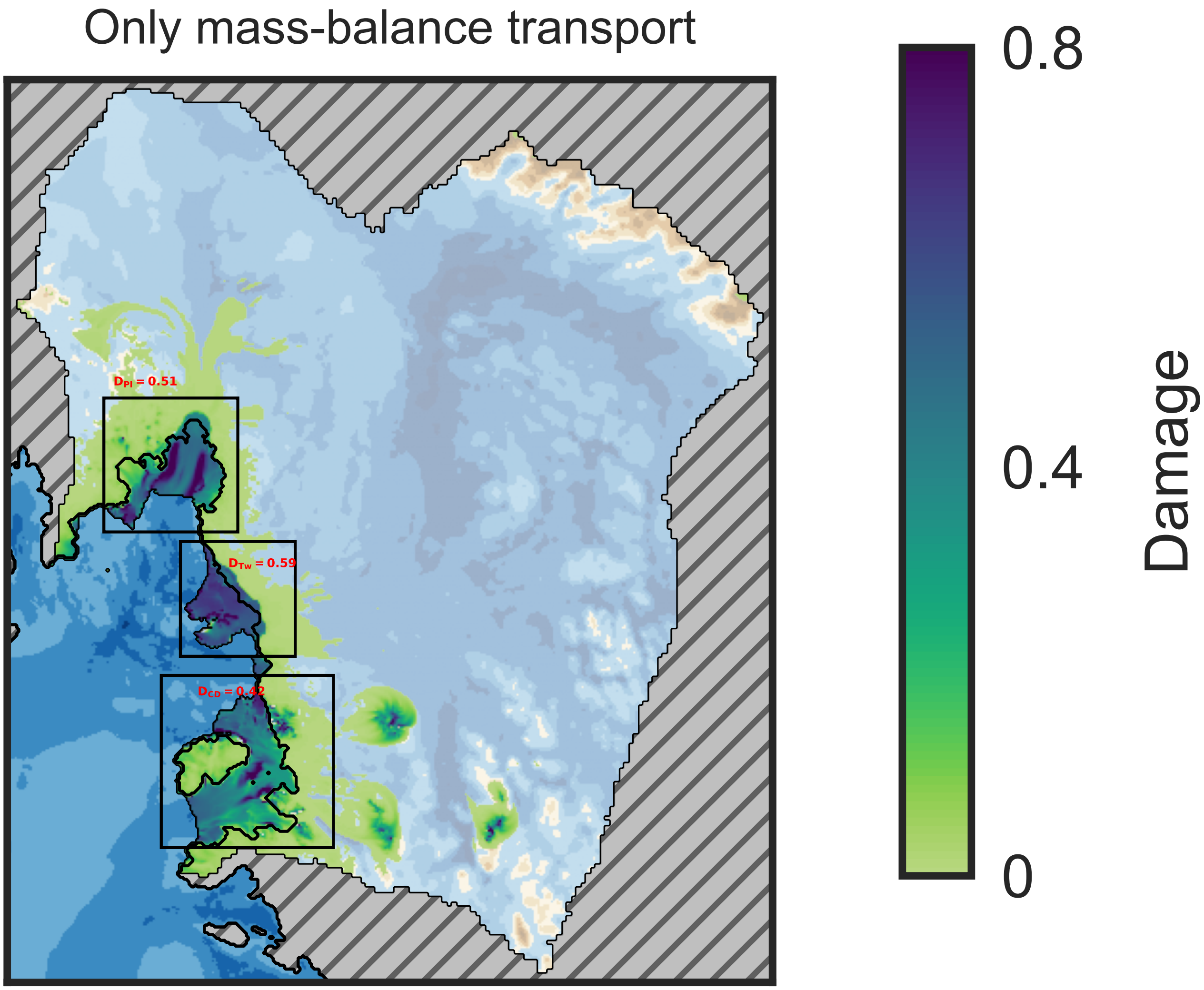

Fig. S2. Removing the long-wavelength limit from the damage transport equation ($S_0 = 1$) substantially affects the simulated patterns. Without this term, damage in ice-shelf interiors increases to 0.51 for Pine Island, 0.59 for Thwaites, and 0.42 for Crosson–Dotson—an increase of approximately 0.1-0.2 relative to the reference case. The long-wavelength limit reduces damage through two mechanisms: crevasse closure in compressive regimes ($S_0 < 0$) and gravitational restoring forces where hydrostatic pressure dominates ($S_0 > 1$).

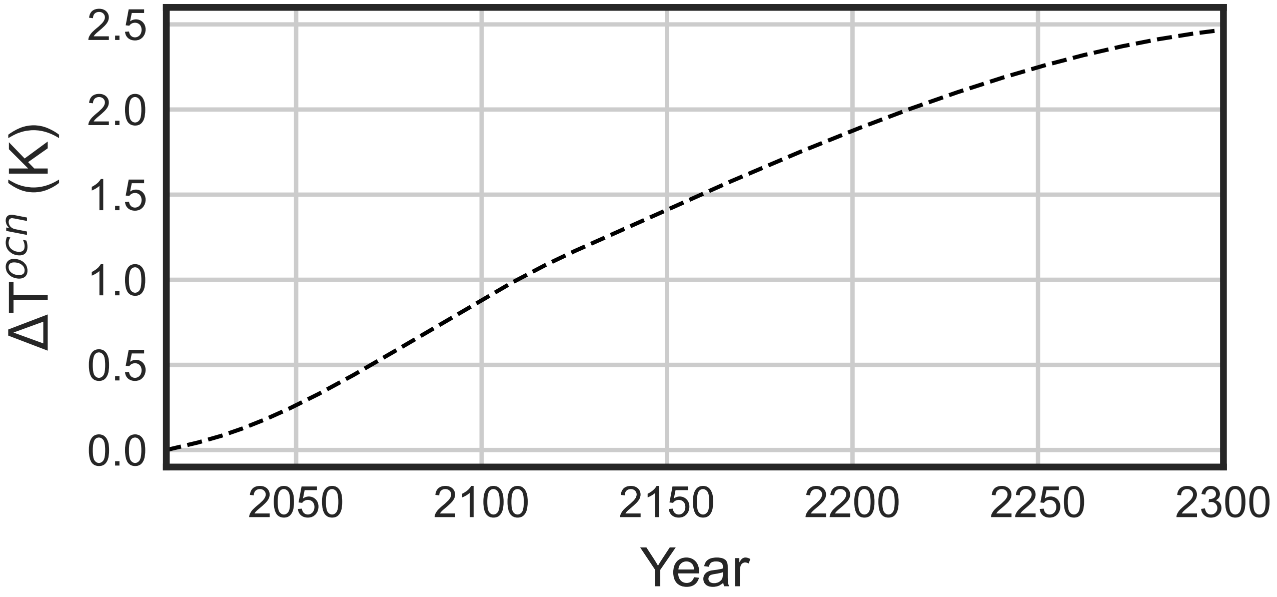

Fig. S3. Homogeneous oceanic forcing anomaly used in this study.

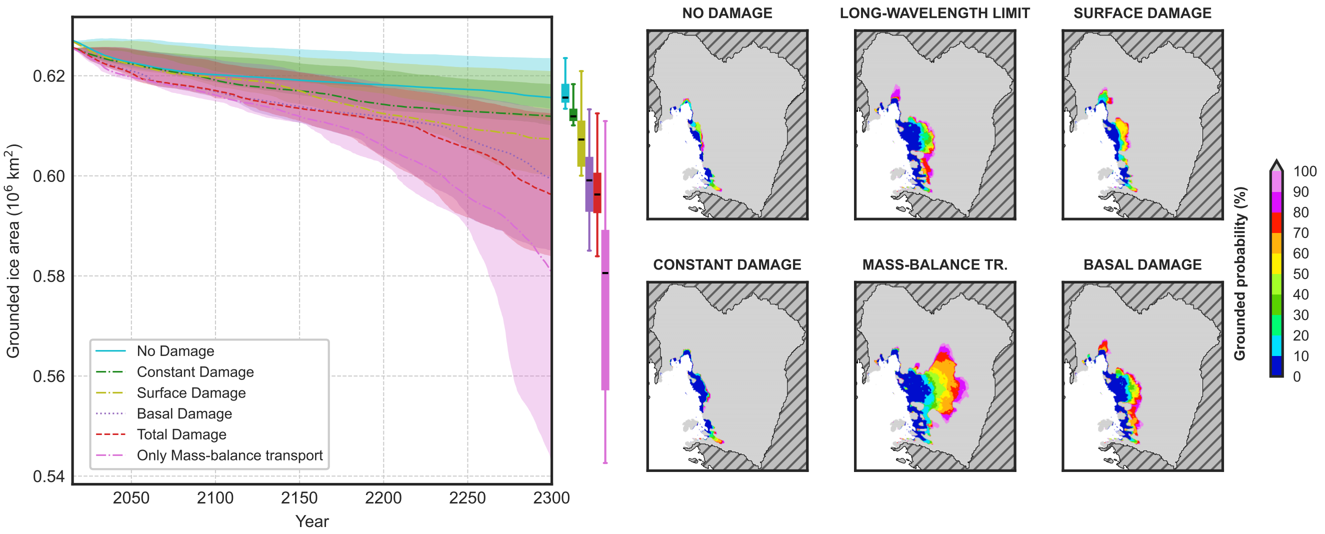

Fig. S4. Left: Simulated grounded ice area for all ensemble members and experiments under the warming scenario. Right: Ensemble grounded-ice probability by year 2300 for each experiment conducted.

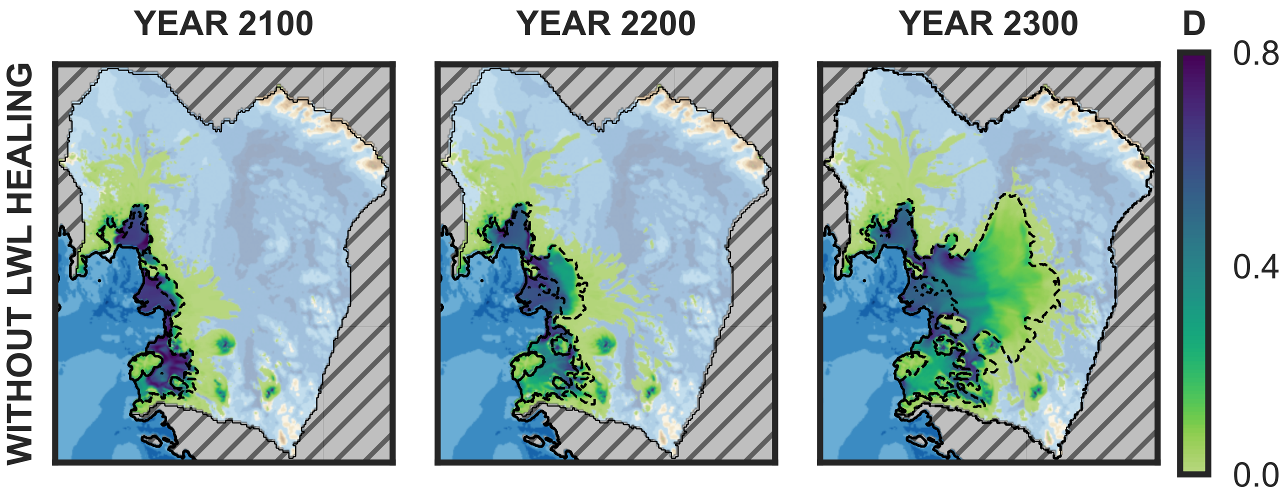

Fig. S5. Snapshots of the mean damage-field evolution at years 2100, 2200, and 2300 for the mass-balance–only formulation.

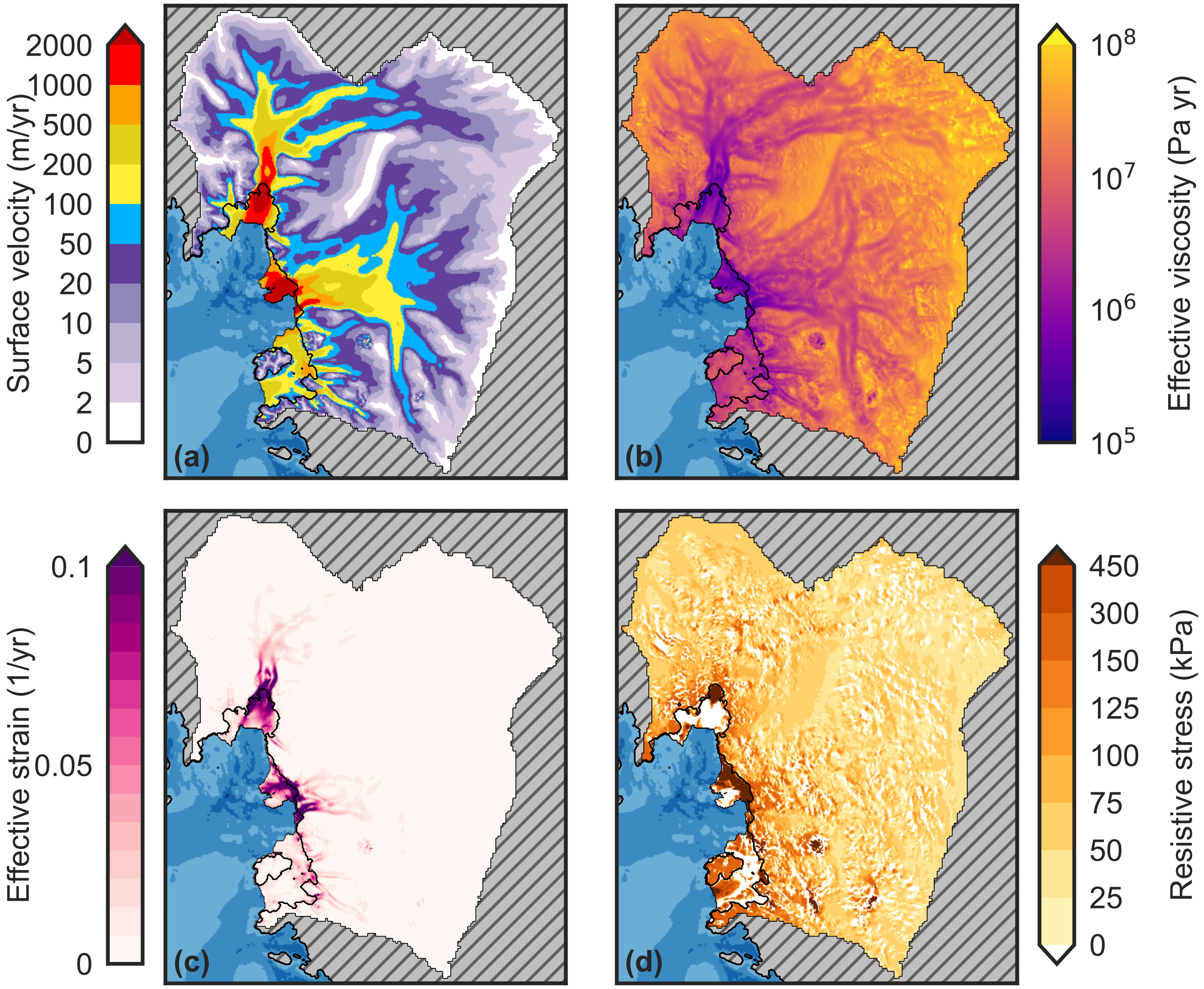

Fig. S6. Simulated present-day fields for the reference case. (a) Surface velocity. (b) Effective viscosity. (c) Effective strain rate. (d) Resistive stress. The reference case includes damage with transport and accounts for the thinning component described in (3).

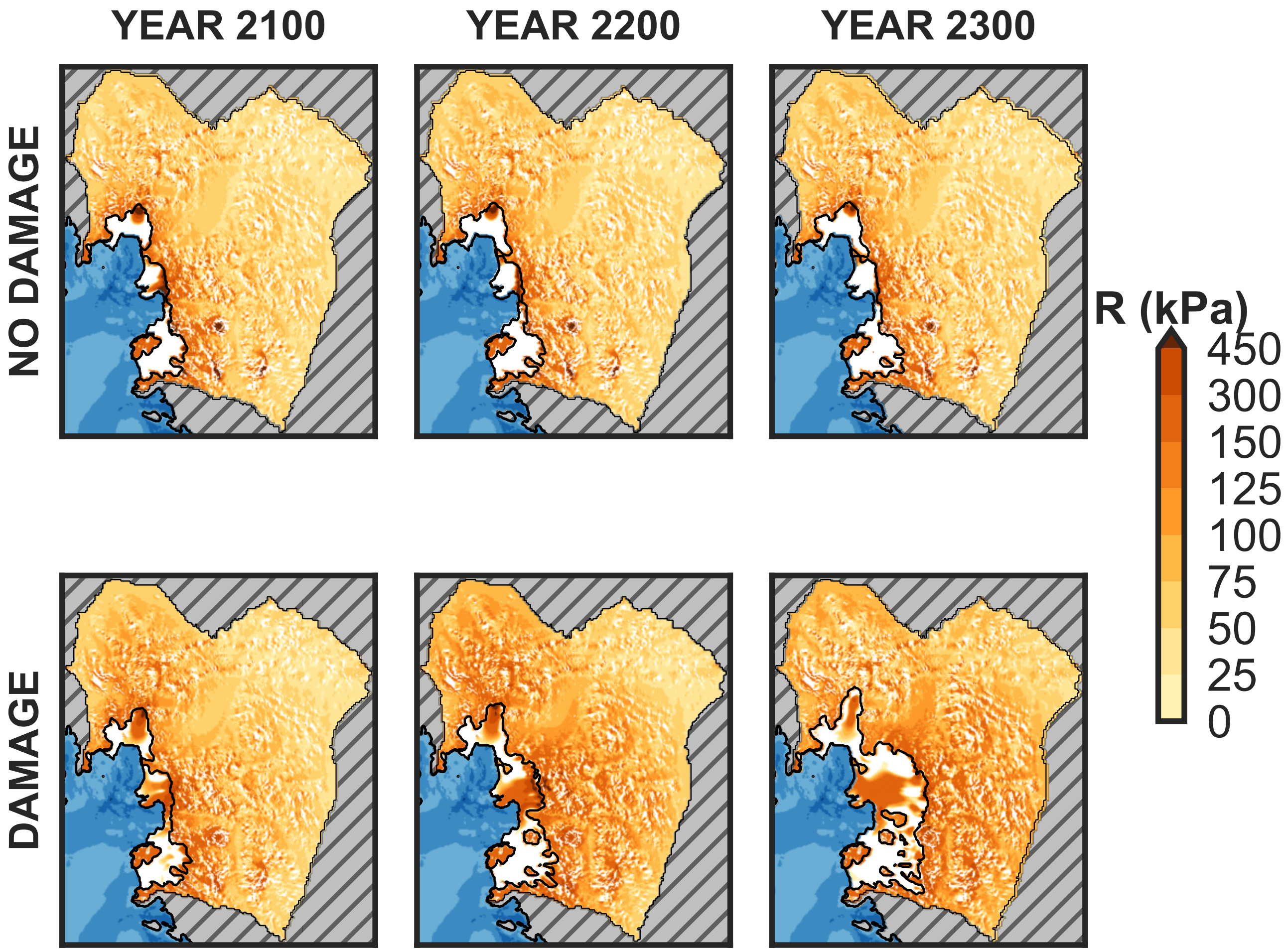

Fig. S7. Resistive stress snapshot for year 2100, 2200, and 2300 for no damage case (upper row) and damage (bottom row) under warming scenario RCP8.5.

Javier Blasco, Violaine Coulon, Maaike Izeboud, Thomas Gregov, Yanjun Li, Frank Pattyn

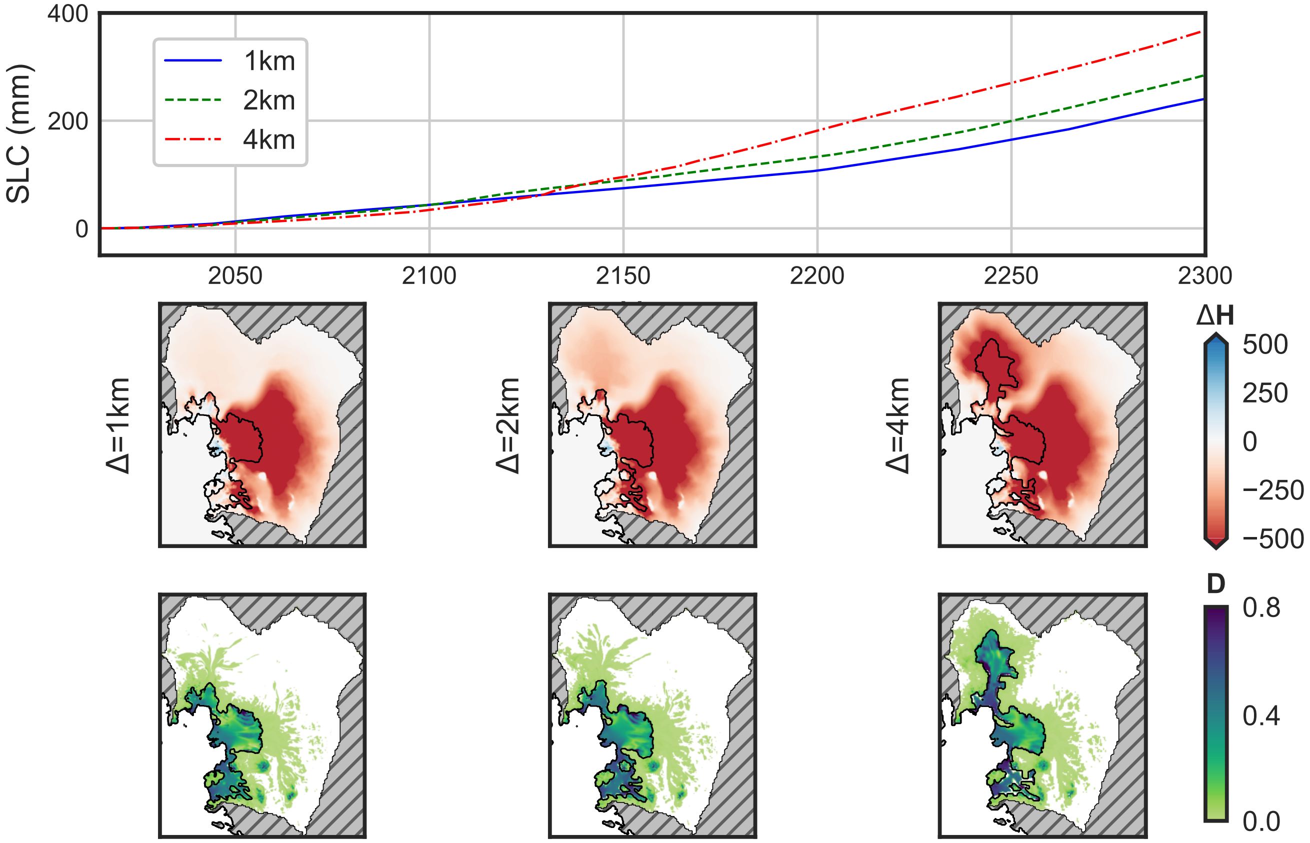

Fig. S8. Upper row: sea-level contribution under the warming scenario for spatial resolutions of 1, 2, and 4 km. Middle row: ice thickness anomaly at the end of the simulation relative to the initial state. Lower row: final simulated damage fields. Solid line represents the grounding-line position.

Scenario	Case	Mean SLR (mm)	IQR (mm)	Ensemble range (mm)
Present-day	Undamaged	22	19–24	0–28
	Fixed damage	34	29–40	3–54
	Evolving damage	78	59–89	12–96
Warming	Undamaged	54	38–67	13–80
	Fixed damage	125	111–132	47–147
	Evolving damage	235	217–259	88–320
Warming	Surface damage	170	141–204	27–222
	Basal damage	207	193–274	72–330
Warming	Mass balance transport	346	293–394	144–460

Table S1. Sea-level rise contribution by 2300 for the Amundsen Sea Embayment under present-day and warming scenarios. Values show the ensemble mean, interquartile range (IQR), and full ensemble range .

Parameter	Units	Values	Description
n	-	3	Glen's flow-law exponent
m	-	3	Friction law exponent
u_0	m yr^{-1}	100-500	Basal velocity regularization term
$\rho_{\rm sw}$	kg m^{-3}	1028	Sea water density
$\rho_{\rm i}$	kg m^{-3}	917	Pure ice density
$L_{\rm i}$	J kg^{-1}	3.34 10^5	Latent heat of fusion ice
$c_{\rm p}$	J kg^{-1} K^{-1}	3974	Specific heat of sea-water
$n_{\rm box}$	-	10	Ice boxes in PICO model
γ_T^*	m s^{-1}	10^{-5}-10^{-4}	Effective turbulent temperature exchange velocity
C	m^6 s^{-1} kg^{-1}	10^6	Overturning strength

Table S2. Table summarizing the model parameters in our experimental setup.

References

1. M Morlighem, et al., Deep glacial troughs and stabilizing ridges unveiled beneath the margins of the antarctic ice sheet. *Nat. Geosci.* **13**, 132–137 (2020).
2. E Rignot, J Mouginot, B Scheuchl, Ice flow of the antarctic ice sheet. *Science* **333**, 1427–1430 (2011).
3. JN Bassis, Y Ma, Evolution of basal crevasses links ice shelf stability to ocean forcing. *Earth Planet. Sci. Lett.* **409**, 203–211 (2015).
